# Effect of Silicon on the Tolerance of Wheat (*Triticum aestivum* L.) to Salt Stress at Different Growth Stages: Case Study for the Management of Irrigation Water

**DOI:** 10.3390/plants7020029

**Published:** 2018-04-03

**Authors:** Daoud A.M., Hemada M.M., Saber N., El-Araby A.A., Moussa L.

**Affiliations:** 1 Soils and Water & Environment Research Institute, Agricultural Research Center, Giza 12112, Egypt; elarabyamira@yahoo.com (E.-A.A.A.); mlobnamy@yahoo.com (M.L.); 2 Botany Department, Faculty of Science, Alexandria University, Alexandria 21511, Egypt; mabroka1999@hotmail.com (H.M.M.); nabilsaber@hotmail.com (S.N.)

**Keywords:** *Triticum aestivum*, salinity, silicate, growth stages, antioxidants

## Abstract

This paper aims to determine the most tolerant growth stage(s) of wheat to salinity stress with the addition of silicon. The aim was to investigate whether saline water could be used instead of good quality water for irrigation without implicating a greater risk to crop production. Local wheat cv. Gimmiza 11 was germinated and grown in sand cultures. Four different NaCl salinity levels were used as treatments: 0, 60, 90 and 120 mM. This was in the presence of 0 and 0.78 mM Si which added as sodium meta- silicate (Na_2_SiO_3_·9H_2_O). Both the NaCl and Si treatments were carried out using a full strength nutrient solution that was adjusted at pH 6.0 and used for irrigation in four replications. The application of Si with the saline nutrient media significantly enhanced superoxide dismutase (SOD) and catalase (CAT) activities in plant leaves at the booting stage compared to the other stages. This was associated with a marked decline in the H_2_O_2_ content. At the booting stage, the Si treatment promoted CAT activity in 120 mM NaCl-stressed leaves compared to the leaves treated with only 120 mM NaCl solution. SOD showed greater prevalence at the booting stage when Si was added into the saline media, and it also revealed maximum activity at the milky stage with salinity stress. This was associated with a smaller reduction in shoot fresh and dry weights, greater reduction in the leaf Na^+^ content and an increase in the K^+^ content, which ultimately increased the cytosolic K^+^/Na^+^ ratio. Chlorophyll *a* and *b* and carotenoid (total photosynthetic pigments) were also higher at the booting stage of salt-stressed plants treated with Si compared to other stages. Accordingly, Si application enhanced the salt tolerance of wheat and reduced the inhibitory effect of Na^+^ and oxidative stress damage as growth proceeded towards maturity, particularly at the booting stage. This shows that saline water can be used for wheat irrigation at the booting stage (much water is consumed) when good quality water is not available for supplemental irrigation. A field study is needed to confirm the greenhouse results.

## 1. Introduction

Wheat is the most important nutritional crop world-wide. The scarcity of irrigation water and salinity build-up are the major constraints on crop production in most arid and semi-arid regions including Egypt.

The deleterious effects of salinity on plant growth are associated with the decrease in osmotic potential in soil solution, nutritional imbalance and specific ion toxicities, particularly Na^+^ and Cl^−^ ions [1]. The negative effects of salinity can also be due to the reduction of the Calvin cycle and CO_2_ intake, which leads to a reduction in chlorophyll content. This causes an overall reduction in the net photosynthesis [2]. Salinity increases the shoot Na^+^/K^+^ ratio that results in the inhibition of metabolic processes [3]. As a result, serious physiological and biochemical changes in the plant occur, causing deterioration in the plant growth and yield [4]. Insufficient time dedicated to the application of saline water and the salinity severity could also be additional reasons for growth retardation [5]. Under stressful biotic and abiotic conditions, the production of reactive oxygen species (ROS) such as superoxide, hydrogen peroxide, hydroxyl radical and singlet oxygen would increase and further attack nucleic acid, photosynthetic pigments, protein and lipids, causing severe damage to the plant cells [6,7]; thus, the ROS could be considered as an indication of stress [8]. Scavenging of the generated ROS is sustained by enzymatic and non-enzymatic antioxidants. Wheat plants increased the antioxidant defense mechanism under abiotic stress to tolerate oxidative damage [6]. The superoxide dismutase (SOD) activity increased to catalyze superoxide conversion to H_2_O_2_ and O_2_ [9]. The generated H_2_O_2_ is converted to water by some antioxidants such as catalase (CAT) and peroxidase (POX), thus eliminating its damage. The antioxidant production rate is related to plant species, genotype and salinity tolerance [1]. However, when a plant faces harsh conditions, the production of ROS will increase and overcome scavenging systems where oxidative stress will burst, causing disturbance in cell metabolism and ultimately cell death [10]. Improving the salt tolerance of plants could decrease the amount of water needed for leaching salts and lead to the disposal of saline water for irrigation at a certain stage of plant growth [11]. Several studies found that the application of silicon could mitigate the hazardous effect of salinity to plants such as wheat [12,13], rice [14], maize [4,15], barley [16] and soybean [17]. Silicon is the second most abundant element after oxygen in soil (28%), but most of its sources are not available to the plant [18]. Silicon exists in soil solution in concentrations that range from 0.1 to 0.6 mM SiO_2_ in the form of monosilicic acid (H_4_SiO_4_) [19] and it is taken up by plants in this form in amounts ranging from >0.1% to about 12% dry weight basis. This depends on the species and genotype of the plant [20]. High accumulation of Si in some halophytic species grown in Qattara Depression in Egypt (range from 2.1% to 5.4%) [21] may be used to support the role of Si in plant salt tolerance. The studies of the interaction between Si and salinity suggested that Si deposition in leaves could limit the transpiration rate and hence improve the water status in cells for better plant metabolism under stressful conditions [22]. In addition, silicon could mitigate the salinity hazard by reducing Na^+^ uptake via the formation of some sort of Na–Si complex in roots [23] or through direct deposition in root cells, causing partial blocking of the transpiration bypass flow [24,25]. Under these conditions, the transfer of K^+^ to the plant tissue would surpass that of Na^+^, causing an increase in the cytosolic K^+^/Na^+^ ratio. This ratio is a key determent of plant salt tolerance [18,26]. In addition, Si maintains chlorophyll content and photosynthetic rate in plants grown under saline conditions [2]. Numerous studies also indicated that Si could alleviate salinity stress by reducing oxidative stress damage and enhancing antioxidant defense system of salt-stressed plants such as maize [27], Barley [28], Canola [29] and wheat [12].

This study characterized the role of Si addition to NaCl-salinized nutrient medium for reducing the salinity stress on wheat growth at different phenological growth stages.

## 2. Materials and Methods

The experiments were conducted in a greenhouse at the Soil Salinity Laboratory in Alexandria. Wheat seeds *(Triticum aestivum* L., cv. Gimmiza 11) provided from the Field Crops Research Institute (ARC) were germinated and grown on 2 December 2015 in acid washed quartz sand (saturation percent, SP 20%). Seven kilograms of sand was packed in plastic pots (25 cm in diameter and 30 cm in height) with an outlet at the bottom for free drainage. The pots were firstly moistened with one-fifth strength nutrient solution (Table 1) for 12 days until seedling establishment.

The seedlings were thinned to keep 30 uniform plants per pot. Thereafter, full-strength nutrient solution was supplemented with 0, 60, 90 and 120 mM NaCl, without or with 0 or 0.78 mM Si that was added as sodium meta-silicate (Na_2_SiO_3_·9H_2_O) was used for irrigation. The nutrient solution was prepared by adding nutrients to tap water instead of distilled water (tap water: electric conductivity 675 μSm^−1^; osmotic pressure −0.35 bar). All of the solutions were adjusted at pH 6.0 and renewed biweekly with an extra amount that exceeded the sand SP by 30%. This was to prevent excessive salt accumulation in the plant root-zone. Plant-shoots were harvested just above the top surface of soil pots at seedling, tillering, booting and milky growth stages, which correspond, respectively, to 22, 43, 80 and 120 days from seedling emergence. The harvested shoots were used for the following determinations.

### 2.1. Growth Parameters, Chlorophyll and Carotenoid Contents

The harvested plant shoots were rinsed thoroughly with distilled water for immediate measurements of fresh weight. Portions of plant materials were oven dried at 70 °C for 48 h for dry weight determinations. Chlorophyll *a* and *b* were measured in fresh fully expanded leaves after extraction with *N*,*N*-Dimethyl formamide [31] using a spectrophotometer (JENWAY, 6705 UV-Vis, Staffordshire, UK) at wavelengths of 647, 665 and 453 nm. Concentrations of Chl.*a* and Chl.*b* were estimated using the formula:

Chl.*a* = 12.7 A_665_ − 2.79 A_647_,

Chl.*b* = 20.7 A_647_ − 4.62 A_665_.

Carotenoids were estimated using the formula [32]:

Carotenoid = 4.2 A_453_ − (0.0264 Chl.*a* + 0.426 Chl.*b*).

### 2.2. Determination of Ions and Protein Contents

Oven-dried and grinded leaves (0.1 g) were digested with a mixture of concentrated H_2_SO_4_ and 30% H_2_O_2_ [33]. Sodium and K^+^ in the digest were determined with a flame photometer (JENWAY, PFP-7, Staffordshire, UK). Protein content was determined in frozen grinded plant materials at 595 nm [34], using a spectrophotometer (JENWAY, 6705 UV-Vis, Staffordshire, UK).

### 2.3. Estimation of the H_2_O_2_ Content

Hydrogen peroxide content in fresh leaves was estimated [35], as follows: fresh leaf tissues (50 mg) were homogenized in ice paths with 5 mL of 1.0% (*w*/*v*) trichloroacetic acid. The homogenate was centrifuged at 12,000× *g* for 15 min. An aliquot of plant extract (0.5 mL) was added to 0.5 mL potassium phosphate buffer (pH 7.0) and 1.0 mL of 1.0 M KI; then, the absorbance of supernatant was measured at 390 nm using a spectrophotometer (JENWAY 6305, Staffordshire, UK). The H_2_O_2_ content was calculated by comparison with a standard calibration curve using different concentrations of H_2_O_2_.

### 2.4. Enzyme Extraction and Activity Assay

Frozen fresh leaves were used to extract SOD and CAT enzymes, as follows: 0.5 g leaf tissues were homogenized in ice cold 0.1 M sodium phosphate buffer (pH 7.5) containing 0.5 mM EDTA, and centrifuged for 15 min at 15,000× *g* [36]. The supernatants were used for the determination of SOD and CAT activities.

SOD activity was estimated by recording the decrease in absorbance of the superoxide nitro blue tetrazolium complex caused by the enzyme. The absorbance was recorded at 560 nm, and one unit of enzyme activity was taken as the quantity of enzyme, which reduced the absorbance reading to 50% as compared with tubes lacking leaf extract [2].

For CAT activity, the reaction mixture contained potassium phosphate buffer (pH 7.0), H_2_O_2_ and leaf enzyme extract. The reaction started by the addition of H_2_O_2_ and the utilization of H_2_O_2_ was recorded at intervals of 30 s by measuring the decrease in absorbance at 240 nm for 3 min [37]. The enzyme activity was computed by calculating the amount of H_2_O_2_ decomposed and expressed as µmol of H_2_O_2_ decomposed g^−1^ fresh weight.

### 2.5. Statistical Analysis

Analysis of variance was performed for the recorded data. The mean values were compared with the least significant differences (LSDs) at 0.05 levels of probability [38]. Standard errors (±S.E.) of means were also calculated and presented in a bar diagram.

## 3. Results

**Figure 1:**
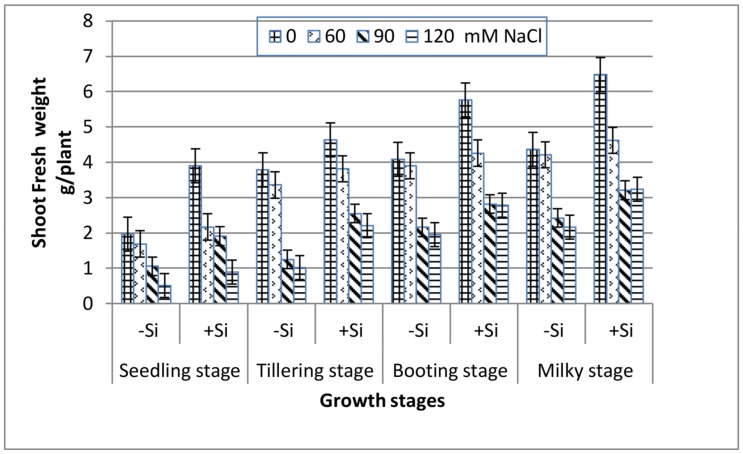
Effect of NaCl on the shoot fresh weight of wheat at different growth stages in the presence and absence of silicon.

**Figure 2:**
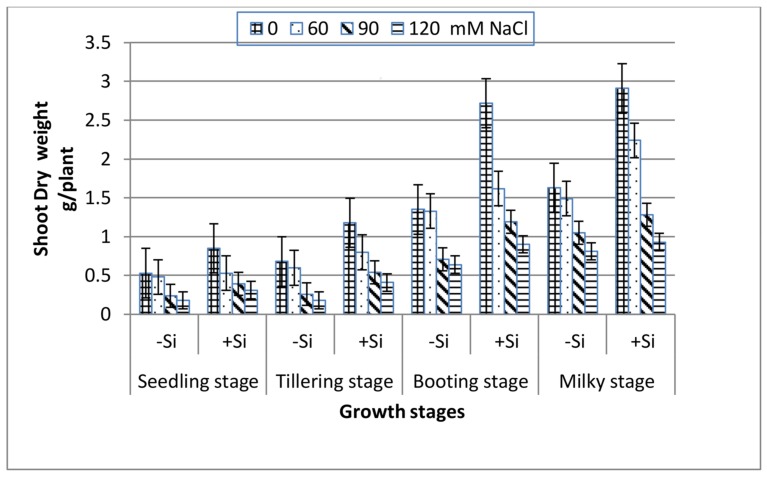
Effect of NaCl on the shoot dry weight of wheat at different growth stages in the presence and absence of silicon.

All of the growth stages of wheat, i.e., seedling, tillering, booting and milky exhibited a gradual decrease in both shoot fresh and dry weight with increasing salinity (Figure 1 and Figure 2). At the extreme salinity (120 mM NaCl), the biomass reductions (fresh or dry) were the highest at seedling and tillering (70%), while booting and milky stages demonstrated lower weight loss (51%) as compared with the control. When Si was added into the saline media, the shoot biomass production enhanced greatly along all of the growth stages. At seedling, tillering, booting and milky stages, the shoot dry weight of 120 mM NaCl-stressed plants in the presence of 0.78 mM Si increased by 1.7, 2.3, 1.4 and 1.1 fold, respectively, over the 120 mM NaCl treatment alone.

**Figure 3:**
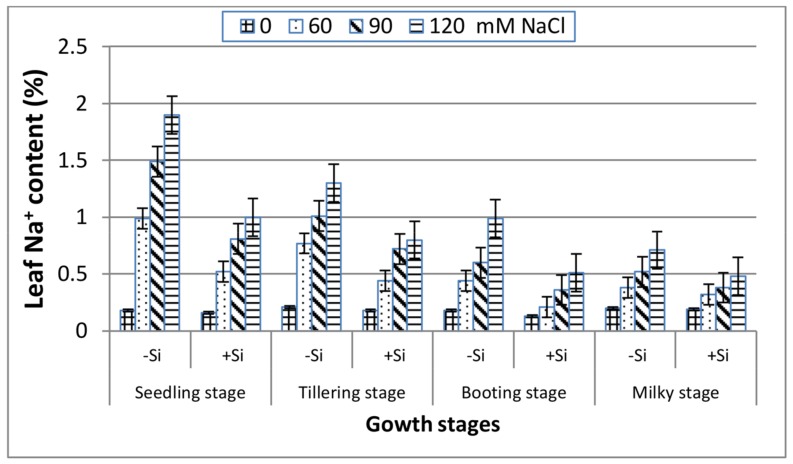
Effect of NaCl on the leaf Na^+^ content of wheat at different growth stages in the presence and absence of silicon.

In the absence of Si (Figure 3), a gradual increase can be indicated in the leaf Na^+^ content with salinity stress along the four growth stages. The most pronounced increase in Na^+^ was recorded at the seedling stage followed by the tillering stage, whereas the other two stages (booting and milky) reacted similarly and showed lower Na^+^ content, indicating their better adaptation to salinity stress. Inclusion of Si into the saline media exhibited a marked decrease in the leaf Na^+^ content along all of the stages in comparison with Si-untreated plants, and that was remarkably great at the booting stage. However, under non-saline conditions, the leaf Na^+^ content was unaffected by Si application at all of the growth stages.

**Figure 4:**
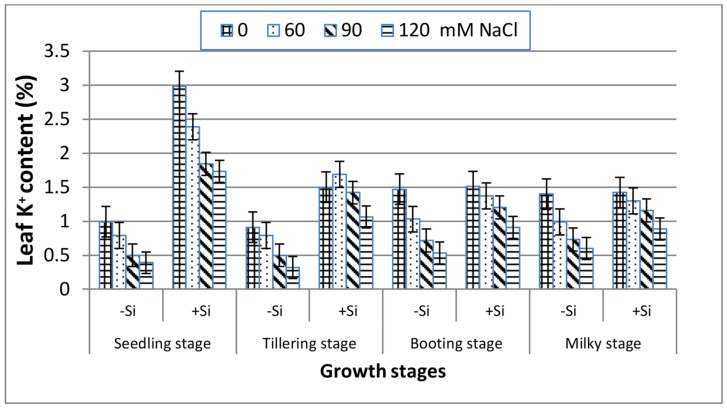
Effect of NaCl on the leaf K^+^ content of wheat at different growth stages in the presence and absence of silicon.

Unlike the trend of the Na^+^ content, K^+^ concentrations in leaves (Figure 4) decreased dramatically with increasing salinity as compared to the control at the four stages. The K^+^ trend was very similar at booting and milky stages and exhibited a lower reduction than seedling and tillering both under stressed and non-stressed conditions. With the application of Si, the concentrations of leaf K^+^ increased remarkably along all of the stages under saline treatments, and this was more pronounced at booting and milky stages.

**Figure 5:**
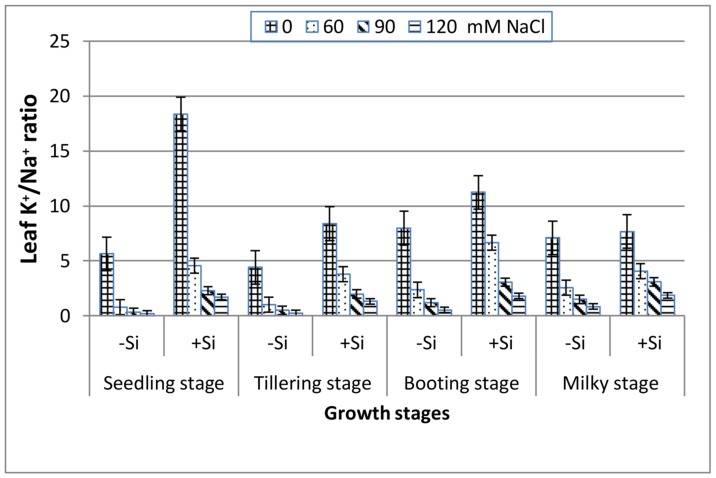
Effect of NaCl on the leaf K^+^/Na^+^ ratio of wheat at different growth stages in the presence and absence of silicon.

As seen in Figure 5, the increase in salinity elucidates a significant reduction in the leaf K^+^/Na^+^ ratio along the four stages. The addition of Si induced a greater leaf K^+^/Na^+^ ratio in the saline media and this was in the following order: booting > seedling > milky > tillering stage.

Chlorophyll *a* and *b* and total carotenoid gradually decreased with salinity stress at all of the stages (Table 2). The reduction was marked at the extreme level of salinity (120 mM NaCl). At lower salinity levels, the concentration of chlorophyll *a* was higher at tillering and booting stages and surpassed that at seedling and milky stages. Meanwhile, chlorophyll *b* was less than chlorophyll *a* at all salinity levels along the four stages. The application of Si increased the total photosynthetic pigments under any given level of salinity during the phenological stages, and the increase was more pronounced at booting as compared with the other stages. These observations, generally, revealed the role of Si in improving the photosynthetic pigments synthesis under salt stress.

It is evident that the H_2_O_2_ content of wheat leaves at the four growth stages was markedly increased in response to the increasing NaCl levels (Table 3). However, the application of 0.78 mM Si to NaCl-stressed nutrient solutions suppressed the H_2_O_2_ content compared to solutions in the absence of Si. The maximum suppression of accumulated H_2_O_2_ was shown at booting and milky stages. The decline in the H_2_O_2_ content in leaves of 120 mM NaCl-stressed plants in the presence of 0.78 mM Si at booting and milky stages was 33% and 31%, respectively, in comparison to those grown in 120 mM NaCl alone.

As seen in Table 4, SOD activity exerted a gradual and significant increase with salinity stress along all of the growth stages as compared with the control. The maximum activity of the enzyme was recorded at the milky stage followed by booting, tillering and seedling stages, respectively. In the non-saline control, however, the enzyme activity was at its maximum at the booting stage. The addition of Si to the saline nutrient media exhibited an additive increase in SOD activity along the four growth stages, where the rate of increase was at its maximum at the booting stage and minimum at the seedling stage. The results also indicated that Si addition had an insignificant effect on SOD activity at the seedling stage under the studied NaCl treatments.

**Figure 6:**
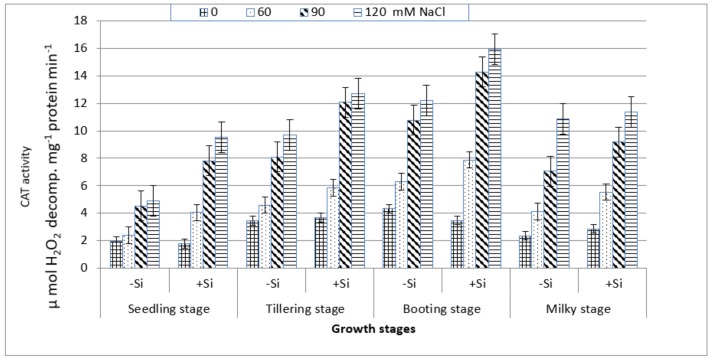
Effect of NaCl on catalase (CAT) activity in wheat leaves at different growth stages in the presence and absence of silicon.

On the other hand, CAT activity increased gradually with the increasing salt stress at all of the growth stages (Figure 6) and this increase was particularly notable at the booting stage followed, respectively, by the tillering, milky and seedling stages. Under non-saline conditions in the presence or absence of Si, the maximum increase in the enzyme activity was also associated with the booting stage followed by the tillering stage. Although the enzyme activity was lowest at the seedling stage, it has shown a considerable increase with salt stress ranging from 23% at 60 mM NaCl to 155% at 120 mM NaCl as compared to the control. The addition of silicon into the saline culture of 60 mM NaCl or higher exhibited a greater enhancement in CAT activity at the four stages, particularly at the booting stage as compared to those of salt-stressed treatments without Si treatment (Figure 6).

## 4. Discussion

Salinity stress is known to disturb cellular activities due to the increase in osmotic stress and the production of reactive oxygen species (ROS), which ultimately causes the suppression of plant growth [39]. This study showed that increasing salinity stress caused a significant reduction in the fresh and dry biomass of wheat at all of the growth stages (Figure 1 and Figure 2), indicating the inhibition of cell division and elongation [40]. However, the reduction in biomass was markedly low at booting and milky stages as compared to seedling and tillering at any NaCl level, suggesting their greater tolerance to salinity stress. These findings are in accordance with those reported for corn plants [41], which showed greater susceptibility to salinity stress at the early vegetative stage than at the late vegetative to early reproductive stage. Thus, the observed decrease in the biomass of wheat plants in response to NaCl treatments might be attributed to the increase in the NaCl uptake (Figure 3), which leads to the generation of toxic ROS which causes the disturbance of the plasma membrane as well as ionic imbalance, and hence suppress metabolic processes and growth [8]. Data in Figure 4 and Figure 5 demonstrated a marked decrease in the K^+^ and K^+^/Na^+^ ratio at all of the growth stages with increasing salinity treatments. This is in line with many authors who stated that ionic imbalance occurs due to excessive accumulation of Na^+^, which reduces the absorption of other mineral nutrients [3,10,42]. The addition of Si into the nutrient saline media improved the fresh and dry matter content of salt-stressed wheat significantly, particularly at the booting stage (Figure 1 and Figure 2). This was accompanied by a decrease in the Na^+^ content (Figure 3) and an increase in the K^+^ content (Figure 4) and thus an increase in the K^+^/Na^+^ ratio (Figure 5), which ultimately led to an improvement in the wheat growth and yield under salt stress [2]. In agreement with these observations, several studies have shown that the addition of Si could protect the growth and yield of many agricultural crops from the toxic effect of salinity stress [43,44,45]. This improvement could be illustrated by reducing the damage effects of accumulated Na^+^ on the plasma membrane integrity via the enhancement of K^+^/Na^+^ discrimination [2], stimulation of gene expression [14] and/or stimulation of the ROS scavenging system [46,47].

On the other hand, NaCl stress is known to suppress the photosynthetic pigments content and photosynthetic machinery in several plants, causing inhibition of plant growth. In agreement with these views, the results in Table 2 show that, in the absence of Si, chlorophyll *a* and *b* contents decreased with the increasing NaCl levels. This suppression might be related to the inhibitory effect of NaCl on chlorophyll biosynthesis [48] and/or the enhancement of chlorophyllase activity as well as the destruction of the chloroplast and thylakoid membrane [49]. With Si addition, chlorophyll *a* and *b* of salt-stressed wheat increased, to some extent, at all of the growth stages (Table 2). These findings suggest that Si offsets the inhibitory effect of salinity by reducing the uptake of Na^+^, enhancing the K^+^/Na^+^ ratio and scavenging generated ROS. The latter was indicated by a decrease in H_2_O_2_ and an increase in SOD and CAT activities, which ultimately resulted in improving the net photosynthesis and growth. The addition of Si was found to increase the tolerance of tomato plants to salt stress through increasing SOD and CAT activities, chlorophyll contents and enhancing the photochemical efficiency of PS II [50]. The alleviation of salinity damage to canola plants was also recorded with Si addition and this was highly correlated with the improvement of chlorophyll contents [51].

Many authors [52,53,54] have reported a marked increase in ROS generation under salt stress and this was accompanied by the suppression of plant growth. In accordance with these views, the H_2_O_2_ content in wheat leaves at the four growth stages significantly increased with increasing NaCl concentrations (Table 3), and this was associated with a marked decline in fresh and dry weights (Figure 1 and Figure 2). This reduction could be attributed to the oxidative stress of H_2_O_2_ and destruction of plasma membranes. Conversely, the addition of Si to saline nutrient medium markedly decreased H_2_O_2_ accumulation, particularly at booting and milky stages, and hence improved the plant growth. These observations could be related to scavenging the generated ROS by enhancing the SOD and CAT activities, thus reducing, to some extent, the inhibitory effect of salt stress.

The data in Table 4 and Figure 6 show that the application of Si gradually increased SOD and CAT activities with increasing salt stress at the booting stage and this was associated with a marked decline in the H_2_O_2_ content and an increase in fresh and dry weights. These observations could reveal the role of Si in the stimulation of antioxidant enzymes activity (SOD, CAT), which reflect the suppression of oxidative stress on the plasma membranes and hence improve the growth under salt stress. However, lower SOD and CAT activities and the greater damage of plasma membranes were found in salt-sensitive than salt-tolerant wheat in the presence of Si [36]. Opposite results were recorded on drought-stressed wheat, where Si did not affect SOD and CAT activities at the booting stage [55]. It seems likely that the effectiveness of the addition of Si into enzymatic antioxidants is not only related to plant genotype, but also to the source of stress.

## 5. Conclusions

In conclusion, the results of this study indicate that the addition of Si into saline nutrient media was effective in alleviating the inhibitory effects of salt stress on the biomass production of wheat, particularly at the booting stage. This was induced by a greater stimulation of the enzymatic antioxidant system, a decrease in H_2_O_2_ generation, an increase in the photosynthetic pigments and improvement of the K^+^/Na^+^ selectivity ratio, which ultimately protected the plasma membrane integrity and functions. Therefore, at the booting stage of Si-treated wheat, saline water instead of fresh water (in case of shortage) can be used for irrigation without implicating a greater risk to crop growth and yield. Field trials are needed to confirm the greenhouse results.

## Figures and Tables

**Table 1:** Composition of full-strength nutrient solution [30].

Compound	Concentration
KNO_3_	3 mM
Ca(NO_3_)_2_	2 mM
NH_4_H_2_PO_4_	0.5 mM
MgSO_4_	0.5 mM
KCl	25 µM
H_3_BO_3_	12.5 µM
MnSO_4_·H_2_O	1 µM
ZnSO_4_·7H_2_O	1 µM
H_2_MO_4_	0.25 µM
CuSO_4_·5H_2_O	0.25 µM
NiSO_4_·6H_2_O	0.1 µM
Fe-EDTA	100 µM

**Table 2:** Effect of NaCl on Chl. *a* and *b* and carotenoid contents (mg g^−1^ fresh weight) of wheat leaves at different growth stages in the presence and absence of silicon.

Treatments		Seedling Stage				Tillering Stage				Booting Stage				Milky Stage			
NaCl mM	Si mM	Chl.*a*	Chl.*b*	Cart.	Total	Chl.*a*	Chl.*b*	Cart.	Total	Chl.*a*	Chl.*b*	Cart.	Total	Chl.*a*	Chl.*b*	Cart.	Total
0	0	9.77	6.72	4.09	20.58	14.55	9.76	6.63	30.9	16.31	8.73	8.73	33.77	10.01	6.07	12.66	28.74
60	0	8.91	6.52	3.91	19.34	12.61	9.17	7.96	29.7	14.52	10.65	8.21	33.38	8.93	4.31	14.31	27.55
90	0	6.62	5.33	3.16	15.11	10.92	8.33	8.23	27.5	9.09	6.95	9.95	25.99	6.71	4.06	11.92	22.69
120	0	4.08	4.91	3.7	12.69	7.08	6.04	8.91	22.0	6.13	4.91	8.91	19.95	4.09	1.71	16.97	20.77
0	0.78	9.82	6.96	3.21	19.99	15.22	10.2	6.69	32.1	16.95	12.24	7.91	37.10	9.52	6.71	11.91	28.14
60	0.78	8.76	6.71	3.62	19.09	15.18	9.76	7.21	32.2	15.67	11.52	7.67	34.86	10.69	6.03	11.36	28.08
90	0.78	8.31	6.01	3.55	17.87	13.23	8.99	7.76	30.0	13.05	8.36	7.51	28.92	7.71	5.88	12.71	26.30
120	0.78	6.21	4.99	4.09	15.19	10.51	8.12	7.09	25.7	9.17	6.11	8.64	23.92	5.12	4.61	12.74	22.47
LSD _0.05_		1.09	1.12	2.18	4.35	2.58	1.93	1.18	2.26	2.71	1.80	2.24	5.19	1.49	2.38	2.19	4.49

Chl. = Chlorophyll; Cart. = Carotenoid.

**Table 3:** Effect of NaCl on the H_2_O_2_ contents (µmol g^−1^ fresh weight) of wheat leaves at different growth stages in the presence and absence of silicon.

NaCl mM	Si mM	Seedling Stage	Tillering Stage	Booting Stage	Milky Stage
0	0	10.1	13.5	19.9	19.8
60	0	18.7	22.8	16.7	18.9
90	0	31.1	50.6	66.4	88.1
120	0	16.9	73.5	89.7	119.7
0	0.78	9.9	16.8	17.1	17.3
60	0.78	12.5	18.4	8.0	9.8
90	0.78	22.1	34.9	53.9	48.9
120	0.78	38.5	54.2	60.3	79.4
LSD _0.05_		4.81	9.11	8.93	7.50

**Table 4:** Effect of NaCl on total protein, T.P. (mg g^−1^ dry matter) and superoxide dismutase (SOD) (Units * mg^−1^ T.P.) activity of wheat leaves at different growth stages in the presence and absence of silicon.

Treatment		Seedling Stage		Tillering Stage		Booting Stage		Milky Stage	
NaCl mM	Si mM	T.P.	SOD	T.P.	SOD	T.P.	SOD	T.P.	SOD
0	0	87.1± 7.3	0.81± 0.06	102.9 ± 8.6	3.34± 0.24	127.9±10.7	6.09± 0.44	136.3 ± 11.4	2.50±0.18
60	0	58.2 ± 6.5	3.43 ± 0.38	83.2±9.2	5.62± 0.62	99.1 ± 11.0	8.11± 0.90	102.1 ± 11.3	4.43 ± 0.49
90	0	40.0 ± 4.0	5.05 ± 0.42	60.9 ± 6.1	9.25 ± 0.77	74.1 ± 7.4	13.0 ± 1.15	83.1 ± 8.3	11.65 ± 0.97
120	0	115.0 ± 8.2	7.21 ± 0.56	133.8 ±9.6	8.45 ± 0.65	148.5 ± 10.6	16.90 ± 1.30	154.4 ± 11.0	17.21 ± 1.32
0	0.78	97.0 ± 10.8	0.84 ± 0.08	128.1 ± 14.2	3.03 ± 0.09	136.9 ± 15.2	5.44 ± 0.22	148.1 ± 16.5	1.91 ± 0.08
60	0.78	69.6 ± 8.0	6.21 ± 0.52	94.1 ± 7.8	8.81 ± 0.73	110.1 ± 9.2	10.31 ± 0.86	113.3 ± 9.4	9.85 ± 0.82
90	0.78	51.1 ± 3.9	7.85 ± 0.87	75.2 ±5.8	13.65 ± 1.52	83.6 ± 6.4	18.09 ± 2.01	87.4± 6.7	20.41 ± 2.27
120	0.78	91.2 ± 8.3	9.47 ± 0.95	116.5 ± 10.6	19.77 ± 1.98	139.7 ± 12.7	22.24 ± 2.22	143.2 ± 13.0	27.97 ± 2.80
LSD _0.05_		-	2.02	-	2.62	-	4.91	-	6.88

T.P. = Total protein, * = See Materials and Methods.
